# Valorization of Glass Fiber Waste (VCAS) as a Precursor in Alkali-Activated Systems Cured at Room Temperature–Influence of SiO_2_/Na_2_O Molar Ratio

**DOI:** 10.3390/ma18184260

**Published:** 2025-09-11

**Authors:** Mauro Mitsuuchi Tashima, Lourdes Soriano, Ester Gimenez-Carbo, José Monzó, María Victoria Borrachero, Jordi Payá

**Affiliations:** 1 Departamento de Ingeniería Minera y Civil, Universidad Politécnica de Cartagena–UPCT, 30203 Cartagena, Spain; 2 Institute of Concrete Science and Technology (ICITECH), Universitat Politècnica de València, 46022 València, Spain; lousomar@upvnet.upv.es (L.S.); esgimen@cst.upv.es (E.G.-C.); jmmonzo@cst.upv.es (J.M.); jjpaya@cst.upv.es (J.P.)

**Keywords:** room temperature curing, alkali-activated systems, glass fiber waste, valorization, microstructure, SiO_2_/Na_2_O molar ratio

## Abstract

Alkali-activated materials are a promising alternative for reducing CO_2_ emissions and raw materials consumption due to their capacity to reuse waste materials. In this study, glass fiber-derived waste (vitreous calcium aluminosilicate, VCAS) is used as a precursor in alkali-activated systems for long curing age at room temperature. Here, the influence of SiO_2_/Na_2_O molar ratio on the mechanical, mineralogical, and microstructural properties is assessed. The XRD pattern, thermogravimetric analysis (TGA), and scanning electron microscopy (SEM) studies demonstrated the evolution of microstructure even after 28 curing days yielding a dense-compact microstructure, and according to the compressive strength results in mortars, about 100 MPa in compression was achieved after 360 curing days for 0.48 and 0.55 SiO_2_/Na_2_O molar ratio, confirming the stability of this system at room temperature.

## 1. Introduction

In recent years, several studies have reported the use of alternative binders for producing mortars and concrete [1,2,3]. This use can be attributed to the necessity of reducing environmental problems associated with Portland cement (OPC) production, which is responsible for 5–8% of global CO_2_ emissions [4]. In this sense, alkali-activated systems emerge as an alternative to OPC due to their comparable mechanical and durability properties. Furthermore, this alternative type of cement is produced by reacting a precursor (amorphous aluminosilicate material, usually fly ash or blast furnace slag) with an alkaline activator (NaOH, KOH, or sodium silicate solution). Depending on the chemical composition of the employed precursor, the hydrated phases formed can be similar to those achieved in the OPC hydration, which means C-(A)-S-H gel. It occurs when calcium is present in the precursor, resulting in the formation of silicates and aluminates chains arranged in layers and separated by layers of Ca(OH)_2_ and interlayered spaces.

The possibility of using waste materials as precursors or even in the production of the alkaline-activating solution (amorphous silica or alkali source) makes the use of alkali-activated systems very attractive from the environmental and economic point of view [5,6]. Nevertheless, the variety of waste materials, their lack of heterogeneity, and their limited availability are the main problems associated with their large-scale usage [7].

Hence, searching for abundant and homogeneous aluminosilicate sources that can be used as precursors in alkali-activated systems could be deemed to be compulsory. One possibility is the development of amorphous precursors with a chemical composition similar to conventional precursors such as blast furnace slag (BFS) or fly ash (FA). In this context, Martín-Rodríguez et al. [7] produced an artificial precursor using chemical reagents (silica gel, aluminum oxide, calcite, and sodium carbonate) by thermal treatment with a chemical composition similar to type C fly ash, rich in calcium compounds. According to these authors, depending on the temperature of synthesis (1000 °C, 1100 °C, and 1250 °C were tested), high amorphous content (about 100%) can be achieved, and the mechanical strength of alkali-activated systems based on the artificial precursor activated with 8 M of NaOH yielded about 70 MPa, achieving similar compression to those systems based on blast furnace slag [7].

Another possibility to achieve abundant and homogeneous aluminosilicate sources is the use of industrial wastes with homogeneous composition. This is the case of the glass fiber industry, where vitrified calcium aluminosilicate (VCAS) with low alkali content is formed by a rapid water quenching of extruded molten material [8,9]. Moreover, its amorphous structure, associated with a high specific surface area and low water demand due to its dense, compact structure, makes its use viable in cementitious systems.

At first, VCAS was tested as a supplementary cementitious material in Portland-blended cements by Shirazi et al. [8]. Some studies report its use, presenting improvements on the slump test of concretes prepared with up to 15 wt.% of VCAS in respect to the control sample. Moreover, the compressive strength of concretes containing VCAS (6–15 wt.%) achieved similar or even higher values for all assessed curing ages (3, 7, 14, 28, and 56 days) compared to the control sample. Neithalath et al. [9] reported an interesting study about VCAS in high-performance cementitious systems. According to the authors, VCAS does not present a cementing effect. Moreover, VCAS contributes to Portland cement hydration (nucleation effect) for up to 7 curing days, and only after this period was the consumption of portlandite in the pastes noted, indicating its pozzolanic reactivity.

In 2012, Tashima et al. [10] assessed the possibility of using vitreous calcium aluminosilicate from glass fiber waste (VCAS) in the production of alkali-activated systems, achieving 70 and 77 MPa in compression for mortars activated with 10 mol.kg^−1^ of KOH and NaOH, respectively. In another study, Tashima et al. [11] compared the effect of thermal curing at 65 °C and room temperature for an alkali-activated mortar based on VCAS for a specific mix proportion (binder: sand ratio 1:3, water/VCAS ratio 0.45, H_2_O/Na_2_O of 11.11 and, SiO_2_/Na_2_O 0.44), achieving similar compressive strength (about 75 MPa) for mortars cured during 90 days at room temperature and mortars cured at 65 °C during 3 days.

This study aims to assess the effect of the chemical composition of the alkaline-activating solution (SiO_2_/Na_2_O molar ratio) on the microstructure and on the compressive strength of alkali-activated systems based on VCAS at room temperature. In addition, the stability of alkali-activated systems based on VCAS were reported for long curing ages at room temperature. The XRD characterization, thermogravimetric analysis, and SEM images of pastes were used to support the compressive strength development of alkali-activated systems based on VCAS up to 360 curing days.

## 2. Materials and Methods

### 2.1. Materials

Vitreous Calcium Aluminosilicate (VCAS) from VitroMinerals (Jackson, TN, USA) is a white pozzolan, generated in the glass fiber industry. According to X-ray fluorescence analysis, VCAS presents, as major components, SiO_2_, Al_2_O_3_, and CaO (see Table 1). Although showing a high amount of CaO (about 23 wt.%), VCAS does not present hydraulic properties [9].

**Figure 1:**
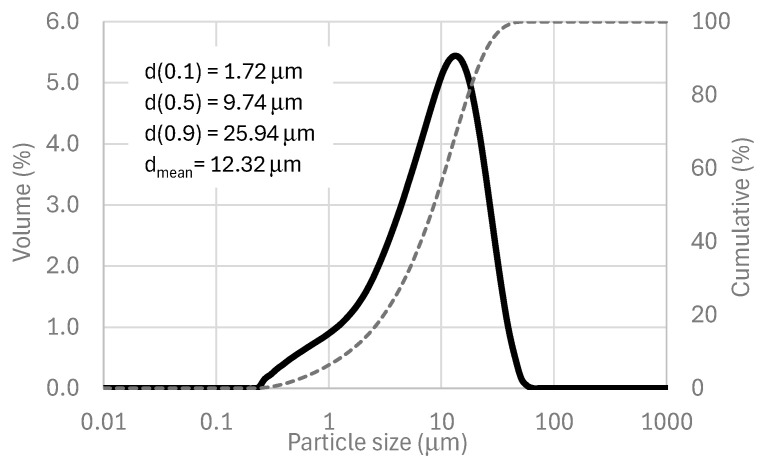
Particle size distribution of VCAS.

**Figure 2:**
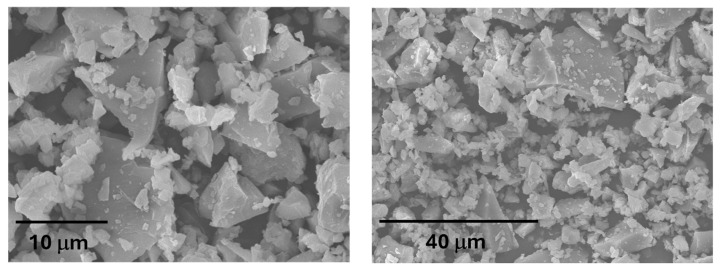
SEM images of the morphology of VCAS particles.

**Figure 3:**
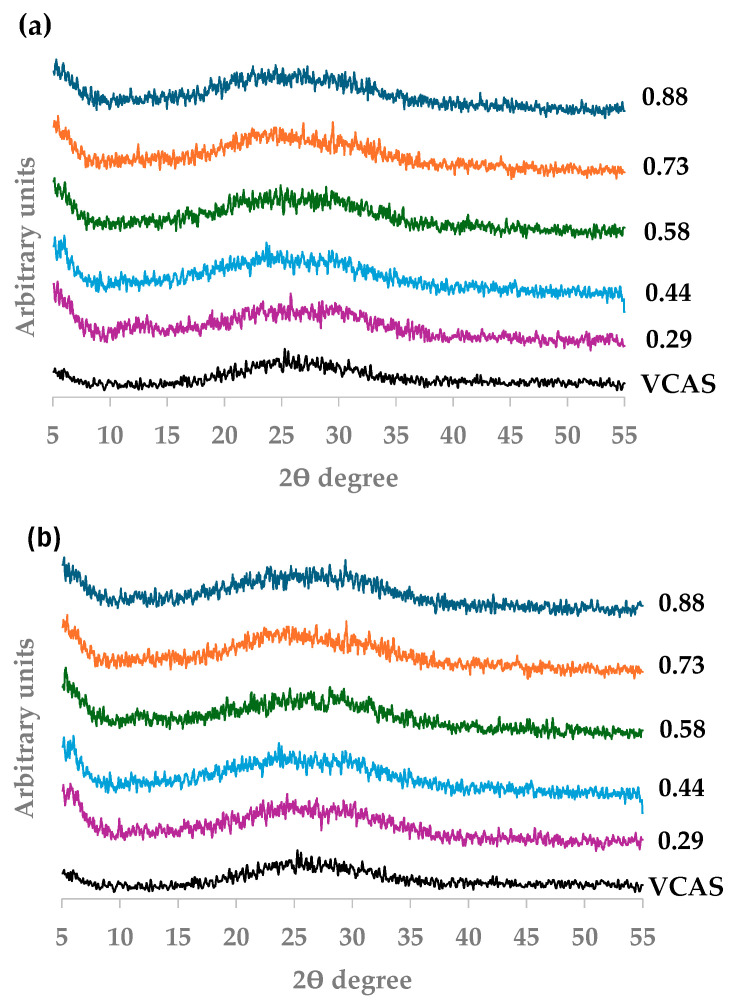
XRD patterns of alkali-activated pastes prepared with different SiO_2_/Na_2_O molar ratios: (**a**) 28 curing days; (**b**) 90 curing days.

Its unimodal particle size distribution (determined using a laser granulometry, Malvern Instruments, Worcestershire, UK, Figure 1), combined with an irregular shape morphology, a dense-compact structure (Figure 2), and its mineralogical (amorphous material) and chemical composition, makes VCAS suitable for its use as an aluminosilicate source (precursor) in alkali-activated systems. The VCAS is an amorphous material characterized by the baseline deviation in 2θ range 15–35°, observed in the XRD pattern (see Figure 3).

Alkaline-activating solutions were prepared dissolving NaOH (98% purity, supplied by Panreac SA, Barcelona, Spain) and Na_2_SiO_3_ solution (28% SiO_2_, 8% Na_2_O, 64% H_2_O from Merck, Madrid, Spain) in tap water. In this context, a siliceous sand with a specific gravity of 2680 kg/m^3^ (determined according to UNE-EN 1097-6 [12]) and a fineness modulus of 4.1 (determined according to UNE 146301 [13]) was also used to produce mortars.

### 2.2. Preparation of Alkali-Activated Systems

In this study, the effect of SiO_2_/Na_2_O molar ratio varying from 0.29 to 0.88 for a fixed H_2_O/Na_2_O molar ratio (11.11) and 10 mol.kg^−1^ of NaOH was evaluated for alkali-activated systems based on VCAS as a precursor while using a water/VCAS ratio of 0.45. The concentration of activator used is based on a previous study [11]. To facilitate the identification (ID) of the samples, they were named according to their SiO_2_/Na_2_O molar ratio: 0.29; 0.44; 0.58; 0.73; 0.88.

The alkaline-activating solution was prepared at least 2 h before it was used to cool down the dissolution temperature. In the production of mortars, the alkaline-activating solution was mechanically mixed with VCAS for 3 min. Then, the siliceous sand was added in a mass proportion of 1:3 binder/sand ratio, and the mortar was mixed for 1 min more. Next, mortars were molded (4 × 4 × 16 cm^3^) in a vibration table for 1 min. After that, molds were covered using a plastic film to avoid water evaporation. After 72 h of curing, the specimens were demolded and sealed in a plastic film. All specimens were cured at room temperature (20 °C, RH > 95%) until the testing age. Pastes composed for microstructural analysis were prepared similarly without sand incorporation.

### 2.3. Tests Performed

The microstructural analysis of alkali-activated pastes based on VCAS was performed using different instrumental techniques. The mineralogical phases of raw material and alkali-activated systems were characterized by using X-ray diffraction (XRD, Philips PW1710, Philips, Tuscaloosa, AL, USA) with Cu Kα radiation under routine conditions of 40 kV and 20 mA, in the 2θ range of 5–55°. Thermogravimetric Analysis (TGA, 850 Mettler Toledo thermo-balance, Mettler Toledo, Barcelona, Spain), to precisely determine the water related to the hydration products, was performed using sealed and pin-holed 100 μL aluminum crucibles in a nitrogen atmosphere (75 mL/min) in the range of 35–600 °C at 10 °C/min heating rate.

The microstructure of fractured pastes covered with gold was assessed through Scanning Electron Microscopy (SEM, JEOL JSM-6300, JEOL, Frenchs Forest, Australia) with 20 kV. For the above analysis, 5–6 small pieces of paste were immersed in acetone for 30 min and then dried in an oven at 60 °C for 30 min to stop the hydration process for each curing age.

Samples for XRD and TG/DTG analysis were manually crushed in an agate mortar with acetone (to stop the hydration process), then dried in an oven at 60 °C for 30 min, and finally sieved through using an 80 μm sieve. Particles smaller than 80 μm were used in the analysis.

The compressive strength of mortars (4 × 4 × 16 cm^3^) cured at room temperature at 28, 90, 180, and 360 curing days was performed using a universal test machine following the procedure described in UNE-EN 196-1 [14].

## 3. Results and Discussion

### 3.1. Characterization of Pastes

The XRD patterns of alkali-activated pastes based on VCAS cured at room temperature containing different SiO_2_/Na_2_O molar ratios are depicted in Figure 3.

Independently of the SiO_2_/Na_2_O molar ratio and curing time, alkali-activated pastes based on VCAS presented an amorphous microstructure characterized by a baseline deviation in the range of 15–35°. No evidence of crystalline phases such as zeolitic structures could be detected, as it usually occurs in alkali-activated systems [15]. Moreover, no significant difference was detected from 28 to 90 curing days. A similar trend was observed for pastes cured at 360 curing days.

In XRD patterns of alkali-activated systems based on blast furnace slag, the presence of some impurities such as quartz, calcite, albite, or even the presence of some crystalline phases formed due to the alkaline reaction such as gehlenite magnesian, calcium silicate hydrate, or magnesium aluminum hydroxide is usually found [16].

In the TG analysis, a continuous mass loss was observed for all assessed samples. Taking into account the DTG curves, a mass loss rate peak around 140 °C is observed for all SiO_2_/Na_2_O molar ratios. According to the literature [15], this peak can be assigned to the dehydration of C-A-S-H gels and/or (N,C)-A-S-H gels formed in the alkaline-activating reaction of aluminosilicates containing calcium. Table 2 shows the total mass loss for all assessed samples for 28, 90, and 360 curing days. For 28 curing days, pastes with lower SiO_2_/Na_2_O molar ratio achieved lower-associated mass loss. This finding confirms the importance of amorphous silica in the formation of hydrated products at early curing times [17].

Comparing the mass loss achieved for pastes after 28 curing days, with samples cured at 90 curing days, an increment in the mass loss is confirmed for all assessed samples. Significantly, this indicates that VCAS continues to react after 28 curing days. The slow reaction of VCAS can be attributed to the dense and compact structure of its particles, which makes its dissolution difficult and, consequently, the alkaline-activating reaction.

**Figure 4:**
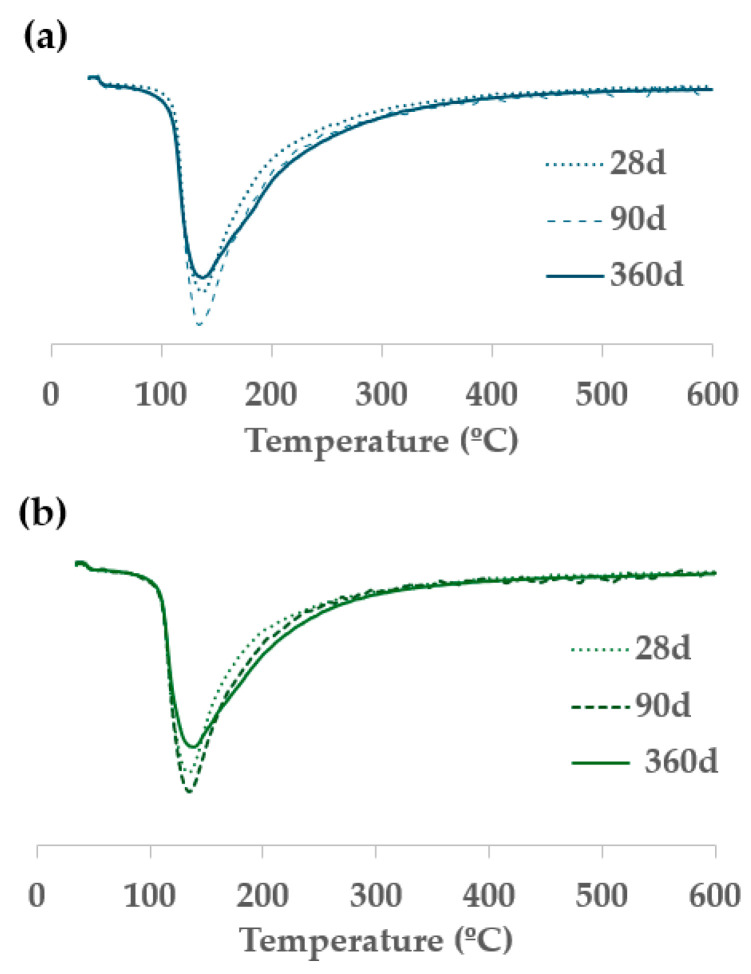
Comparative analysis of DTG curves of alkali-activated pastes cured at 28 and 360 days: (**a**) paste 0.44; (**b**) paste 0.58.

Furthermore, from 90 to 360 days of curing, a slight reduction in mass loss is observed with respect to 90 days of curing. This behavior can be attributed to rearranging the microstructure in alkali-activated systems based on VCAS during prolonged curing periods. When comparing the DTG curves of samples cured at 28, 90, and 360 days, a notable increase in the shoulder at higher temperatures becomes evident. As an example, Figure 4 depicts the DTG curves of pastes 0.44 and pastes 0.58 cured at 28, 90, and 360 days, indicating that bonded OH^−^ groups and/or combined water are more strongly linked to the C-A-S-H/(N,C)-A-S-H gels (increment in the shoulder) after 360 days compared to 28 days.

**Figure 5:**
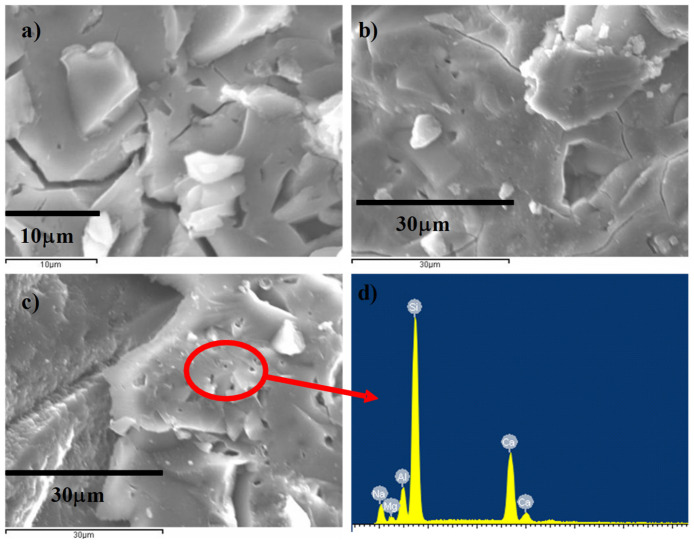
SEM images of alkali-activated pastes cured at 28 days: (**a**) paste 0.29; (**b**) paste 0.58; (**c**) paste 0.88; (**d**) EDX of paste 0.88.

According to the selected SEM images of alkali-activated pastes based on VCAS, no significant differences can be crucially detected due to the variation in the SiO_2_/Na_2_O molar ratio (Figure 5). For all pastes cured at 28 days, a dense, compact microstructure is observed, and several VCAS particles can be seen embedded in the gel matrix. This finding indicates that VCAS is not easily dissolved in an alkaline medium, a fact that justifies the continued reaction observed in thermogravimetric analysis from 28 to 360 curing days. As seen in the EDX analysis (Figure 5d), the hydrated products formed are mainly C-A-S-H gel and (N,C)-A-S-H gel.

**Figure 6:**
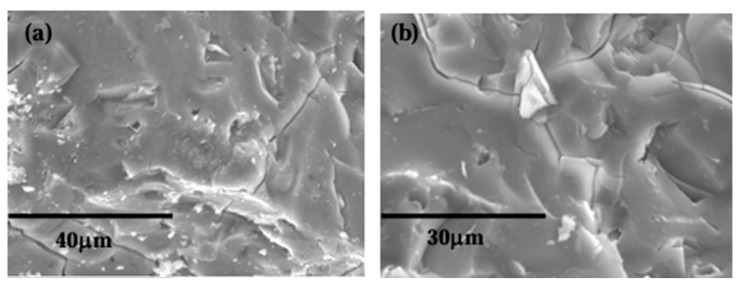
SEM images of alkali-activated pastes cured at 360 days: (**a**) sample 0.44; (**b**) paste 0.73.

After 360 curing days (Figure 6), fewer VCAS particles embedded in the cementitious matrix were observed. Vitally, this indicates the continuous reaction of VCAS over time. The microstructure of alkali-activated systems based on VCAS is dense and compact, similar to those observed after 28 curing days. Comparing the microstructure of alkali-activated systems based on VCAS with alkali-activated systems based on blast furnace slag, a significant difference was observed as follows: alkali-activated systems based on blast furnace slag present a microstructure similar to Portland cement, a more porous structure with some crystalline phases [15].

### 3.2. Compressive Strength of Mortars

**Figure 7:**
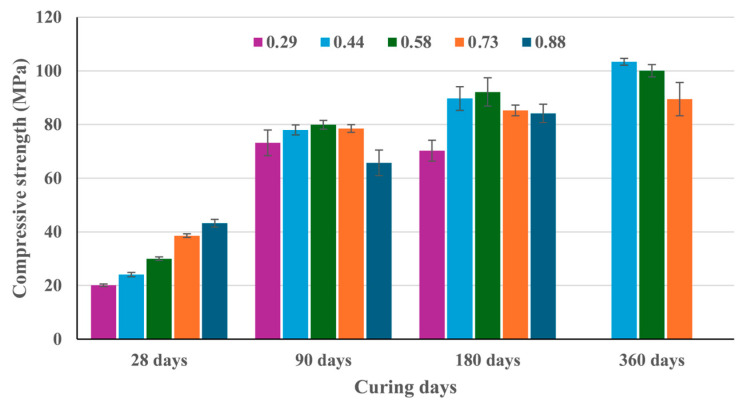
Compressive strength development of alkali-activated mortars based on VCAS using different SiO_2_/Na_2_O molar ratios.

Figure 7 shows the compressive strength development of alkali-activated mortars based on VCAS for different SiO_2_/Na_2_O molar ratios. The obtained results showed the stability of alkali-activated systems based on VCAS when cured at room temperature.

For 28 curing days, increments in the SiO_2_/Na_2_O molar ratio are associated with an enhancement in compressive strength, yielding values from 20.1 MPa to 43.3 MPa. As reported in the literature, the presence of soluble silica at early ages enhances the compressive strength of alkali-activated systems [17]. It occurs due to the rapid exchange and oligomerization reaction between the precursor and soluble silica [18]. For 90 curing days, an expressive increment was yielded when compared to samples tested at 28 days. In this case, most of the samples achieved about 78 MPa. After 360 curing days, samples 0.44 and 0.58 achieved compressive strength values higher than 100 MPa. Comparing the results after 360 curing days to those obtained after 28 curing days, an increment of over 429% was reached.

Hence, the optimal SiO_2_/Na_2_O molar ratio, associated with the maximum compressive strength, is attributed to the mixture 0.44 of SiO_2_/Na_2_O. The existence of an optimum SiO_2_/Na_2_O molar ratio, related to the maximum compressive strength, was also reported in the literature and depends on the type of precursor, curing conditions, etc. [18,19].

Natural logarithm (*ln*) fitting ($CS\;=\;a*\ln\left(x\right)+b$) was performed for mixtures containing a SiO_2_/Na_2_O molar ratio of 0.44, 0.58, and 0.73 to describe the compressive strength (*CS*) development of alkali-activated mortars based on VCAS cured at room temperature in the 28–360 days curing period (*x*). Table 3 summarizes the calculated parameters (*a* and *b*).

Parameter *b* represents the theoretical value of CS for 1 curing day, being significantly higher for the highest SiO_2_/Na_2_O molar ratio (0.73). However, parameter *a* is much higher for the 0.44 SiO_2_/Na_2_O molar ratio (30.932), which means a notable contribution of VCAS activation for longer curing time, despite the lower silicate content in the activator.

According to the obtained results, the reactivity of VCAS particles is much more evident for long curing ages due to their physical properties (dense and compact particles, etc.) [8,9].

## 4. Conclusions

This study has demonstrated the viability of using VCAS as a precursor in alkali-activated systems cured at room temperature. The main specific findings from this study are summarized as follows:

- Alkali-activated systems based on VCAS present stability when cured at room temperature.
- The compressive strength development of alkali-activated mortars indicates the gradual reaction progress of VCAS. It can be associated with the dense and compact structure of VCAS.
- The highest compressive strength of 102 MPa was achieved after 360 curing days for a mixture using the activator with the 0.44 SiO_2_/Na_2_O molar ratio.
- The total mass loss, associated with the dehydration of hydrated compounds such as C-A-S-H gel and (N,C)-A-S-H gel, presented an increment for up to 90 curing days. After 360 curing days, although the mass loss was reduced, the DTG curves indicated that bonded OH^−^ groups and/or combined water were more strongly linked to the gel. This fact justifies the increment in compressive strength up to 360 curing days.
- No evidence of the formation of crystalline phases was detected, independently of the SiO_2_/Na_2_O molar ratio used in the activating solution.
- The SiO_2_/Na_2_O molar ratio played an important role in the development of alkali-activated systems cured at room temperature, achieving an optimal for 0.44 SiO_2_/Na_2_O molar ratio.

## Figures and Tables

**Table 1:** The chemical composition of VCAS is determined by XRF (wt.%).

SiO_2_	Al_2_O_3_	CaO	Na_2_O	MgO	K_2_O	Fe_2_O_3_	Others	LOI
57.90	12.92	23.51	0.74	2.88	0.13	0.47	1.20	0.25

**Table 2:** Total mass loss of alkali-activated pastes based on VCAS using different SiO_2_/Na_2_O molar ratio for different curing ages.

ID	28 Days	90 Days	360 Days
0.88	16.15	18.20	17.33
0.73	15.83	17.58	17.17
0.58	16.00	18.53	18.25
0.44	15.38	18.23	17.66
0.29	15.68	17.36	15.79

**Table 3:** Parameters obtained for logarithmic fitting.

ID	a	b	R^2^
0.44	30.932	−72.458	0.94
0.58	27.733	−55.583	0.92
0.73	20.121	22.163	0.88

## Data Availability

The raw data supporting the conclusions of this article will be made available by the authors on request.
